# The Current Role of Osteoclast Inhibitors in Patients with Prostate Cancer

**DOI:** 10.1155/2018/1525832

**Published:** 2018-12-26

**Authors:** Diomidis Kozyrakis, Dionyssios Paridis, Stefanos Perikleous, Konstantinos Malizos, Anastasios Zarkadas, Antonios Tsagkalis

**Affiliations:** ^1^Department of Urology, “Achillopouleio” General Hospital of Volos, Magnesia, Greece; ^2^Department of Orthopaedics, Animus-Kyanous Hospital, Larisa, Greece; ^3^Department of Orthopaedics, Faculty of Medicine, School of Health Sciences, University of Thessaly, Larissa, Greece

## Abstract

**Purpose:**

Prostate cancer (PCa) is one of the most frequently diagnosed malignancies worldwide. Hormonal deprivation therapy is a well-established treatment for locally advanced or metastatic diseases but exposes patients to the risk of osteoporosis and fragility fractures. Furthermore, the tropism of the PCa cells to osseous metastases increases the incidence of skeletal-related events (SREs).

**Methods:**

A nonsystematic review of the international literature was performed in respect to the use of osteoclast inhibitors zoledronic acid (ZA) and denosumab (DEN) in PCa patients.

**Results:**

DEN and ZA have proved their efficacy in preventing osteoporosis and bone mass loss in patients treated with hormonal therapy with no proven superiority of one agent over the other. However, the effectiveness in reducing fragility fractures has been proved only for DEN so far. In metastatic-free castrate-sensitive high-risk PCa patients, ZA has not shown any efficacy in preventing osseous metastasis, and evidence is lacking in favor or against the use of DEN. The use of osteoclasts inhibitors had no evident positive effect in overall and disease-specific survival in this group of patients. In advanced castrate-refractory malignancy, DEN has shown clinical superiority over ZA in preventing new SRE but not in overall survival.

**Conclusion:**

Superiority of DEN over ZA has been proved only in advanced castrate refractory disease in terms of preventing new SRE. In the rest of the cases, the selection of either agent should be based on the clinical condition of each patient and the cost of the treatment.

## 1. Introduction

Prostate cancer (PCa) is the second most prevalent malignancy worldwide [1, 2]. According to the US Cancer Statistics, during the year 2014, 172,258 new cases were diagnosed and 28,343 deaths were attributed to the disease in this country [1]. Despite the efforts for cancer control, about 6% patients will eventually develop skeletal metastasis during follow-up. This rate climbs up to 80%, should a poorly differentiated PCA is left untreated [3–7].

One of the characteristics of the PCa cells is their tropism to osseous metastases. Bone metastasis is a major factor of morbidity and mortality, causing a plethora of symptoms, such as persistent skeletal pain, bone fractures, spinal cord compression, and severe disability [3, 8, 9] thus, jeopardizing the patients' quality of life (QoL). The term *skeletal-related events* (SREs) has been used as a clinical endpoint which include the detection of pathologic bone fractures, the spinal cord compression, and the need for orthopedic surgery intervention or for palliative radiation to the bone [10].

Not only it is the malignancy itself but also the relevant therapy that may have a negative impact to the skeletal health. Osteopenia and osteoporosis are potential side-effects of pharmaceutical or surgical castration, which pose a threat to the skeletal health. The risk of therapeutic-induced bone fracture is directly related to the duration of treatment [8, 11, 12].

The phenotype of the metastatic PCa is characterized by intense osteoblastic activity. However, recent evidence suggests that a sequence of osteoblastic and osteoclastic activity takes place. At early stages, skeletal metastases with osteolytic lesions are detected but later on, the osteoblastic phenotype prevails at a rate up to 90% [9]. The tumor-induced disruption of the normal bone structure leads to the debulking of the osseous tissue, which explains the fragility of the bones with metastatic sites and the high fracture rates. [9].

Patients with metastatic disease describe episodes of extreme pain as a result of metastatic tumor invasion into the periosteum, the bone marrow cavity, and the bone matrix, which are innervated by sensory and sympathetic fibers. The bone sites affected by the tumor produce and release a plethora of stimuli to the peripheral nerve endings (for example, TNF*α*, prostaglandins (PGs), interleukin-1 (Il-1), and nerve growth factor). The stimuli activate the afferent nerve terminals inducing the phosphorylation of afferent sensory pathways and the generation of action potential. The action potential is transmitted via the primary afferent sensory nerves to the dorsal horn of the spinal cord, which subsequently activates the second-order neurons, thus transmitting the noxious signal up to the somatosensory cortex of the brain. In chronic pain conditions, the peripheral nerves along with other sites in the afferent pathways are shifted to a hypersensitive state and pain is perceived at a lower threshold of noxious stimuli [9].

For addressing the metastatic lesions to the bones and the relevant complications, several therapeutic options have been proposed. These comprise the delivery of external radiation therapy, radiation emitting isotopes (Str-89, Sa-153, and recently Ra-223), chemotherapy, new generation hormonal agents (e.g, enzalutamide), CYP17A1 inhibitor (abiraterone), and bone-targeted agents against osteoclast activity (bisphosphonates and denosumab (DEN)) [3, 13].

It is considered that the bisphosphonates and the RANK-L inhibitors play an important role in preserving the bone health and in inducing the osteoclasts' apoptosis. The rationale behind the use of these agents in osseous metastases is that, by remodeling the osseous tissue, the production of stimuli of the peripheral nerves (TNF, Il, PG, etc.) is controlled and the activation of afferent pathways is minimized [9].

The aim of this review article is to present the contemporary role of bisphosphonates and DEN not only in preventing drug-related osteoporosis and osteopenia but also in alleviating the metastatic pain and in treating the SRE in patients with prostate cancer.

## 2. Materials and Methods

**Figure 1:**
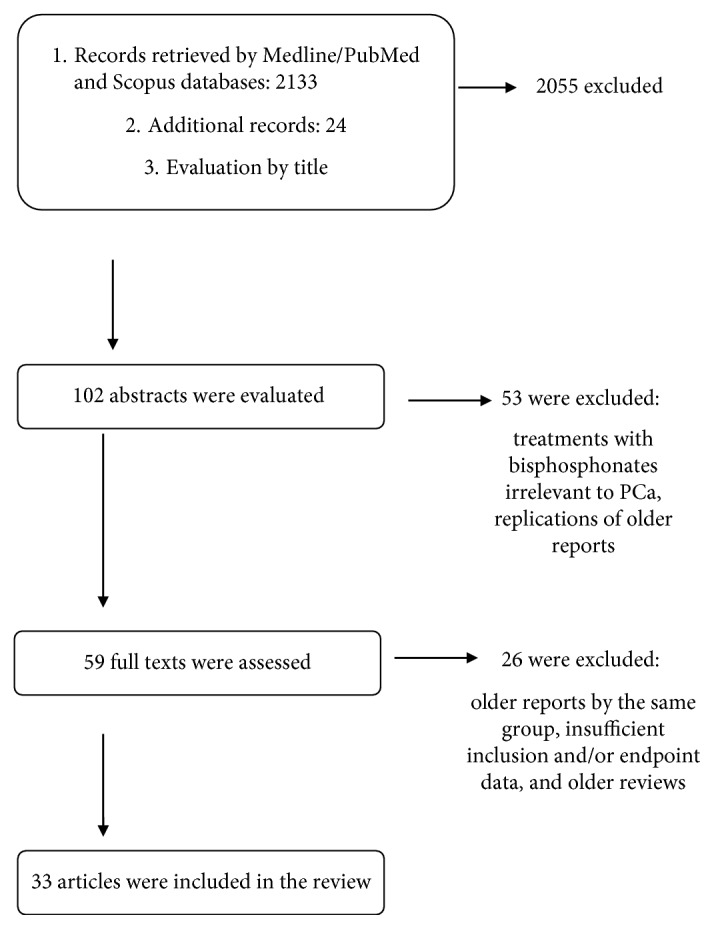
Flowchart of the review process.

A search in Medline/PubMed and Scopus databases from 1980 till now was performed using the following key words: *prostate cancer*, *bone metastasis*, *zoledronic acid*, *denosumab*, and *bisphosphonates*. Additional articles were identified by using the “similar articles” search tool of the Medline electronic platform, as well as by reviewing the list of references of related articles. A list of 2133 titles of articles and 24 additional records were initially evaluated by 2 authors DK and DP for relevance, and 102 abstracts of all types of articles (reviews, randomized controlled trials, and case series) were further examined. Full texts were retrieved by 57/102 articles and, after having the consensus of all the co-authors, 31 of them were included in the study (Figure 1). The clinical effects of osteoclast inhibitors were examined in various prostate cancer subpopulations (for example, castration-sensitive, castration refractory, and with or without skeletal metastasis), and the relevant results are presented herein.

## 3. Results

### 3.1. Bisphosphonates

#### 3.1.1. Pathophysiology

Pyrophosphates are natural anions produced by the hydrolysis of ATP in cells. The biochemical role of these unstable anions is to inhibit the cascade of cholesterol biosynthesis in the osteoclasts' cytoskeleton, promoting changes in its function which induce the apoptosis of the cells [14]. It is estimated that the clinical role of this action is to maintain the integrity and correct the bone mass loss in sites affected by neoplasmatic cells [8]. By substituting oxygen with carbon, a stable compound is generated named bisphosphonate. First generation bisphosphonates are etidronate and clodronate, while pamidronate and zoledronic acid (ZA) are second and third generations respectively [3].

#### 3.1.2. Bisphosphonates (excluding ZA), Castration-Sensitive M1

In older studies performed in castration-sensitive patients with osseous metastases, oral clodronate may marginally (not statistical significantly) prolong the onset of new skeletal symptoms and marginally increase survival compared to placebo. The drug is likely to prevent the worsening of the performance status but with a cost of high incidence of gastrointestinal side-effects, increased lactate dehydrogenase serum levels, and need for frequent dose adjustments [15]. An update of the original paper published six years later, denoted that first generation bisphosphonates compared to placebo could have a positive impact on overall survival among patients with metastatic disease (HR: 0.77, 95% CI: 0.60–0.98, *p*=0.032). On the contrary, metastases-free patients do not experience prolonged survival (HR: 1.12 95% CI: 0.89–1.42, *p*=0.94) [16].

Other reports revealed that clodronate failed to show clinical efficacy in alleviating pain and improving QoL in patients with castration-resistant disease and osseous metastasis [17]. Administration of 1400 mg clodronate once daily was recently compared with 4 mg of ZA iv once a month. In this prospective randomized trial conducted in castration-sensitive PCa patients with bone metastasis, ZA showed longer bone progression-free survival, improved control of skeletal pain, and significant increase of bone mineral density of the lumbar spine compared with clodronate. However, no benefit in overall survival between the two agents was recorded [18].

Older studies have tested low (30 mg every 2 weeks and 60 mg every 4 weeks) versus high (60 mg every 2 weeks or 90 mg every 4 weeks) dosages of pamidronate, a second generation bisphosphonate, in palliative treatment of metastatic prostate and breast cancer patients. In breast cancer population, a 25% healing of metastatic lesions was observed but only for high dose of pamidronate. In PCa patients, however, none of the tested dosages was effective. It could be assumed that these two malignancies may have a different degree of metastatic potential to the bones at least at baseline [19]. Likewise, Coleman et al. did not reveal any clinical efficacy in high-dose pamidronate in patients with as high bone resorption rates as those encountered in prostate cancer [20].

More recently, in a pooled analysis of 2 randomized controlled trials, Small et al. failed to show any significant improvement of pamidronate in alleviating bone pain and preventing the onset of new SRE in patients with osseous metastasis compared to placebo [21].

#### 3.1.3. ZA, Castration-Sensitive M0

The third generation of bisphosphonates has been extensively examined in several clinical scenarios. In RTOG 0518 phase III clinical trial conducted in castration-sensitive nonmetastatic PCa patients, ZA showed a protective role in preventing hormonal deprivation-induced osteoporotic events compared to placebo but no difference in prevention of bone fractures or in improving QoL [22]. In another study, in which patients exposed to castration treatment for up to one year were recruited, the authors concluded that 4 mg of ZA iv every 3 months statistically significantly increased the bone mineral density by 3.6% in the femoral neck, 3.8% in the total hip, and 6.7% in the lumbar spine over placebo [23]. However, the authors provide no data in respect to fragility fracture rates, and the effects of ZA in fracture risk cannot be estimated.

In the Zometa European Study (ZEUS, 2015), the role of ZA in the prevention of skeletal metastasis in high-risk patients was examined. High risk was defined as PSA equal to or greater than 20 ng/ml and/or positive lymph nodes and/or Gleason score 8–10. Following the administration of the agent every three months for 4.8 years along with the standard therapy, no protective role against bone metastasis was revealed compared to the standard care alone (bone metastases rates: 14.7 and 13.2% for ZA and control group, respectively, *p*=0.65) [24].

#### 3.1.4. ZA, Castration-Sensitive M+

In the STAMPEDE trial (2016), high-risk PCa patients with and without osseous metastases were enrolled. The addition of ZA to the standard care also failed to improve overall survival (OS), and the authors concluded the agent has no role in delaying the onset of metastases in high-risk population [25].

The GALGB 90202 trial studied the effect of early administration (before the onset of castration resistance) of 4 mg of ZA every 4 weeks versus placebo in castration-sensitive metastatic PCa. Similar to the STAMPEDE trial, ZA was associated neither with a prolonged time before the onset of SRE (HR: 0.97, 95% CI: 0–1.17, *p*=0.39) nor with an improved survival (HR: 0.88, 95% CI: 0.70–1.12, *p*=0.29) [26]. In another multicenter randomized phase III trial (ZAPCA), no benefit was demonstrated in respect to the time of treatment failure, the time to new SRE, and OS. However, in patients with low baseline PSA (<200 ng/ml), a beneficial role of ZA in delaying the treatment failure was revealed (HR: 0.58, 95% CI: 035–0.93, *p*=0.023) [27]. In another paper, an improvement in progression-free survival, skeletal pain, and SRE was recorded only in patients with Gleason score equal or greater than 8 [28], while others revealed a beneficial role of ZA only in time to PSA failure [29].

#### 3.1.5. ZA, Castration-Resistant M+

Older studies have demonstrated the beneficial role of the agent in metastatic castration-resistant patients. Saad et al. showed that the administration of 4 mg every three weeks significantly reduced the incidence of SRE (33.2% versus 44.2% of placebo, 95% CI; −20.3% to −1.8%, *p*=0.021), increased time prior to the onset of skeletal events (not reached for ZA 4 mg, 321 days for placebo, *p*=0.011), and reduced the pain scores as well as the need for analgesic consumption compared to placebo. On the contrary, no difference was noted in disease progression, performance status, or QoL. This study also defined that due to the better tolerance profile and fewer side effects, 4 mg is the optimal dose compared to 8 mg [30]. Two years later, an update of the original paper was published, evaluating the long-term results of the agent. Compared with placebo, the administration of 4 mg of ZA was associated with fewer patients with (at least one) SRE (38% vs 49%, 95% CI: −20.2% to −1.3%, *p*=0.28), increased time to first SRE (448 for ZA vs 321 for placebo, *p*=0.009), and reduced the total risk for SRE by 36% (risk ratio 0.64, 95% CI: 0.485–0.845, *p*=0.002), proving the effectiveness of ZA on a long-term basis [31].

Recent evidence suggests that ZA can be effectively combined with docetaxel-containing chemotherapy. Data from the TRAPEZE trial revealed that although no improvement in progression-free survival (PFS) and OS was noted, ZA increased the time interval before the onset of new SRE and diminished the incidence of SRE by approximately one third. The authors concluded that ZA might play a role in maintenance therapy following the chemotherapy [32].

In a recent systematic review based on the Cochrane Controlled Trials Registry and the Medline database, the authors extracted results from 18 randomized controlled trials about the role of bisphosphonates in patients with bone metastasis. They concluded that they had no benefit in pain response and that they probably decreased the disease progression and the number of SRE compared to the control group. However, the clinical benefit should be weighed against the potential complications of the therapy before decision-making [33]. An overview of the main publications regarding ZA is presented in Table 1.

#### 3.1.6. Complications

In respect to the drug-related complications, bisphosphonates, particularly ZA, have been associated with renal failure and jaw osteonecrosis. It was estimated that the rate of renal failure was approximately 15% and 21% for the dose of 4 and 8 mg of ZA, respectively. By adjusting the dose in 4 mg and lowering the infusion rate, the incidence of renal failure reached the corresponding rate of placebo group [30]. The osteonecrosis of the jaw, which is defined as “the presence of exposed bone in the oral cavity despite the appropriate management for 8 weeks,” represents a rare complication with an incidence up to 1.3%. It has been correlated with a history of tooth extraction, poor oral hygiene and perhaps smoking, diabetes mellitus, anemia, and the use of antiproliferative agents [12]. Improving the patients' dental health may diminish the jaw osteonecrosis events. Hypocalcemia is rarely encountered and should be corrected with administration of calcium supplements and vitamin D. Other potential complications are nausea, vomiting, fatigue, hyperpyrexia, anemia, and myalgia [12, 33].

### 3.2. Denosumab

#### 3.2.1. Pathophysiology

Osteoblasts are bone cells that produce new osseous tissue and also express the receptor activator of nuclear factor-κΒ ligand (RANK-L). This molecule binds to the corresponding receptor (RANK) carried by the premature osteoclasts (preosteoclasts). The activation of this receptor promotes the maturation of the cells towards multinucleated osteoclasts. In high turnover conditions, such as in metastatic sites of prostatic malignancy, the RANK-L is highly expressed leading to bone resorption [9, 11]. Denosumab (DEN) a fully human monoclonal antibody IgG2 against RANK-L. It is estimated that the inhibition of osteoclasts' activity promoted by this agent may prevent the bone destruction process and the SRE caused by the deposition and activation of malignant cells on the bone tissue [9].

#### 3.2.2. Castration-Sensitive M0

In 1,468 patients with castration-sensitive PCa without skeletal metastasis, it has been shown that the administration of 60 mg DEN subcutaneously every 6 months for 36 months increased the bone mineral density in multiple skeletal sites (lumbar spine, total hip, and distal radius) [11]. In a comparative study with similar population, the administration of 60 mg DEN statistically significantly decreased the likelihood of new vertebral fractures (RR: 0.38, 95% CI 0.19–0.78; *p*=0.006) compared to placebo [34].

These studies indicate that DEN plays a protective role in preventing osteoporosis and osteopenia, which is frequently diagnosed in patients treated with hormonal deprivation agents. A recent meta-analysis compared the efficacy of antiosteoporotic medications in metastatic-free castration-sensitive patients. DEN and ZA showed equivalent efficacy in terms of improvement of bone mass density [35]. In this meta-analysis, the bone mass density was used as a surrogate of the fracture risk. However, the clinical effectiveness of DEN and ZA in reducing the fragility fracture rates cannot be actually evaluated in this meta-analysis.

#### 3.2.3. Castration-Resistant M0

In a multicenter, randomized double-blind placebo-controlled trial, the role of DEN was evaluated in the prevention of skeletal metastasis in castration-resistant PCa. Compared to placebo, 120 mg DEN administered subcutaneously increased significantly the bone metastasis-free survival by 4.2 months (HR: 0.85, 95% CI: 0.73–0.98, *p*=0.028) and the time to first SRE (HR: 0.84, 95% CI: 0.71–0.98, *p*=0.032) but not the overall survival (HR: 1.01, 95% CI: 0.85–1.20, *p*=0.91) [36]. This study was one of the 6 RCTs included in a recent meta-analysis conducted in nonmetastatic patients. In the rest 5 trials, bisphosphonates were compared with the standard of care and a total of 5,974 cases were analyzed. The polled analysis showed that the bisphosphonates do not alter the incidence of skeletal metastases (HR: 1.09, 95% CI: 0.84–1.41, *p*=0.51). In contrast, the aforementioned DEN trial [36] proved the effectiveness of RANK-L agents in delaying the onset of skeletal lesions in castration-resistant patients. It was also reconfirmed that the use of osteoclasts inhibitors of either group had no effect in overall and disease-specific survival [10].

It should be noted that evidence is lacking regarding the role of DEN in preventing the skeletal metastases castration-sensitive patients. In this group, it has already been shown that ZA does not improve time to SRE and overall survival and cannot be considered as an adjunct to the standard treatment [24, 25]. DEN plays a protective role in the prevention of osteoporosis and in the elimination of vertebral fracture events attributed to the castration. However the role of RANK-L agents in the prevention of skeletal dissemination of malignancy in castration-sensitive high-risk for metastases patients is still unknown and should be addressed in future studies.

#### 3.2.4. Castration-Resistant M+

Other studies have directly compared the clinical effectiveness of bisphosphonates (specifically of ZA) with DEN in castration-refractory PCa patients. In a phase 3 multicenter randomized double-blind study, DEN was superior to ZA in preventing new SRE. The mean time to new (on study) SRE was 20.7 and 17.1 months for DEN and ZA, respectively (HR: 0.82, 95% CI: 0.71–0.95, *p*=0.0002) [37]. In another phase 3 study published in 2015, it was reconfirmed that DEN is superior to ZA in reducing the risk of the first and any subsequent adverse event (*p*=0.005 and *p*=0.004, respectively) associated with the skeletal system, including skeletal pain and subclinical fractures [38].

Others have evaluated the value-for-money of DEN with that of ZA. DEN administration is related to fewer SRE and lower SRE cost. However, the estimated drug-related cost in the US was more than 10,000$ over that of ZA. Likewise, despite the fewer SRE attributed to DEN, the total cost per patient was more than 7,000$ higher than that of bisphosphonates, raising severe questions in respect to the cost-effectiveness of DEN [39]. Publications regarding the use of DEN in prostate cancer patients are provided in Table 2.

#### 3.2.5. Complications

The safety profile of DEN is different from that of bisphosphonates. RANK-L agents have no detrimental effect on the renal function, should GFR is greater than 30 ml/min. Dose adjustment and monitoring of renal function is necessary only in patients with severe renal impairment [14]. Jaw osteonecrosis and hypocalcemia are rare but well-recognized side-effects of DEN encountered at rates up to 5% and 2%, respectively [36]. The risk for osteonecrosis is related to the duration of treatment [12, 14]. Correction of dental pathology and improvement of oral hygiene prior to the initiation of antiresorptive treatment may eliminate the osteonecrosis events. The hypocalcemia is more frequently diagnosed with DEN than with ZA (13% vs 6% in metastatic castration-resistant patients). Administration of oral calcium supplements with vitamin D is recommended for the correction of low calcium levels [12]. Acute phase reactions (e.g., fever, chills, and myalgia) may be encountered in patients under any antiresorptive treatment, but the corresponding rates are lower for DEN (8%) versus ZA (18%). Ocular side-effects, including eyelid edema, cataract, scleritis, and conjunctivitis, attributed to DEN, are very rare and respond well to treatment with cortisone. Cataract has been associated with disorders in calcium homeostasis [14].

## 4. Conclusions

Both main antiresorptive agents have proven their safety and effectiveness in preventing bone mass loss and osteoporosis in patients treated with hormonal therapy. In this population, a face to face comparison of DEN with ZA did not reveal superiority of any regimen over the other. However, effectiveness in reducing fragility fracture risks has been proven only for DEN. In castration-sensitive high risk for metastases PCa patients, ZA has not shown any efficacy in preventing osseous metastasis but DEN has not been extensively evaluated in these patients. Regarding cases with advanced disease in castration-resistant malignancy, DEN has shown clinical superiority over ZA in preventing new skeletal events and complications, but not in overall survival. One study indicates that clodronate may prolong survival in metastatic PCa, but before any generalization, the results should be replicated by others in comparative studies.

Chemotherapy has now emerged as a first-line treatment in patients with metastasic disease, but the combined treatment with docetaxel and ZA did not show any benefit in clinical progression and in overall survival compared to chemotherapy alone. However, the results of the addition of RANK-L agents to chemotherapy have not been examined so far. Furthermore, because none of the osteoclasts inhibitors has been coadministered with the newer hormonal agents, such as enzalutamide or abiraterone acetate in PCa patients, future trials could investigate the clinical outcomes of the combined therapy in metastatic population.

The cost of RANK-L agents, which is very high compared to that of bisphosphonates, should be considered prior to initiation of treatment. In patients with renal impairment, DEN should be preferred over ZA due to better safety profile. There some evidence that jaw osteonecrosis rates are comparable between these two agents and increase with the duration of treatment, but the incidence of hypocalcemia is higher in DEN and acute phase reactions are more frequent with ZA.

## Figures and Tables

**Table 1:** Main publications regarding ZA.

Reference	Study population	Treatment	End points
[22] RTOG 0518	CS M0	ZA 4 mg vs placebo	↑ BMD in lumbar spine (6% vs −5%, *p* < 0.0001), left total hip (1% vs −8%, *p* < 0.0002), and femoral neck (3% vs −8%, *p*=0.0007) No prevention of bone fractures or QoL
[23]	CS M0, <1 year ADT	ZA vs placebo	↑ BMD in femoral neck by 3.6% (*p*=0.0004), hip by 3.8% (*p* < 0.0001), and spine bone by 6.7% (*p* < 0.0001)
[24] ZEUS 2015	PSA>20 ± LN+ ± gleason 8–10	ZA 4 mg + SOC vs SOC	Bone metastases: 14.7% in ZA vs 13.2% in placebo (*p*=0.65); no protection from skeletal metastases
[25] STAMPEDE	CS, high-risk PCa (61% M1, 15% N+/M0, 24% N0M0,	ZA 4 mg + SOC vs SOC vs SOC + Doc vs SOC+ ZA +Doc	Median OS: NR for ZA + SOC vs 71 m for SOC vs 81 m for Doc + SOC vs 76 m for SOC + Doc + ZA No increased survival for ZA No delay of metastases for ZA
[26] GALBG 90202	CS M+	ZA 4 mg vs placebo	Similar time for new SRE: median time: 31.9 m for ZA vs 29.8 m for placebo (*p*=0.39); no increased OS (HR: 0.88; 95% CI: 0.70–1.12, *p*=0.29)
[27] ZAPCA	CS M+	ZA 4 mg + complete androgen blockade (CAB) vs CAB alone	Similar time to failure: 12.4 m vs 9.7 m (HR: 0.75, 95% CI: 0.57–1; *p*=0.051); ↑ time to new SRE for ZA + CAB (64.7 vs 45.9 m, HR: 0.58; 95 % CI 0.38–0.88; *p*=0.009) For PSA < 200, delay in treatment failure for ZA + CAB: 23.7 m vs 9.8 m (HR: 0.58, 95% CI: 0.35–0.93; *p*=0.023)
[30]	CR M+	ZA 8 mg vs ZA 4 mg vs placebo	SRE for 4 mg ZA: 33.2% vs 44.2% placebo, 95% CI; −20.3% to −1.8%, *p*=0.021; SRE for 8 mg ZA: 38.5% vs 32.74% placebo, 95% CI; −15.1% to 3.6%, *p*=0.222. ZA 4 (but not 8) mg: ↓ incidence of SRE, ↑ time to new SRE, ↓ pain score No difference in PCa progression, PS & QoL
[31] (long term results of [30])	CR M+	ZA 4 mg vs placebo	↓ pts with SRE (38% for ZA vs 49%, 95% CI: −20.2% to −1.3%, *p*=0.28), ↑ time to first SRE (448d for ZA vs 321, *p*=0.009), ↓ risk for SRE by 36% (risk ratio: 0.64, 95% CI: 0.485–0.845, *p*=0.002)
[32] TRAPEZE	CR M+/45% radiation therapy, median PSA: 146 mg/ml	Doc vs Doc + ZA 4 mg vs Doc + ZA + Strontium-89 (Sr)	Similar CPFS for Doc vs Doc + ZA: HR, 0.98; 95% CI, 0.85–1.14; *p*=0.81; similar OS for Doc vs Doc + ZA: HR, 0.99; 95% CI, 0.84–1.16; *p*=0.91
[33] meta-analysis	18 RCT, CR M+	ZA vs control	Probably ↓ disease progression and ↓ SRE, with ZA, no effect in mortality, no effect in pair response Concern for potential complications

**Table 2:** Main publications regarding DEN.

Reference	Study population	Treatment	End points
[11]	CS M0	DEN 60 mg vs placebo	↑ BMD in lumbar spine by 7.9%, total hip 5.7%, and distal radius 6.9% (*p* < 0.0001 for all sites)
[34] HALT	CS M0	DEN 60 mg vs placebo	↓ incidence of vertebral fractures at 36 months (1.5% vs. 3.9%) (RR: 0.38; 95% CC, 0.19 to 0.78; *p*=0.006)
[35] meta-analysis	CS M0	DEN vs ZA vs others	Similar improvement in BMD for DEN and ZA (3% hip, 3% femoral, 6% lumbar)
[36]	CR M0/high risk (PSA > 8 mg/ml or and PSA DT < 10 m)	DEN 120 mg vs placebo	↑ median bone metastasis-free survival by 4.2 m with DEN (29. 5 vs 25.2, HR: 0.85, 95% CI 0.73–0.98, *p*=0.028) ↑ time to first SRE (median 33.2 vs 29.5 m; HR 0·84, 95% CI: 0.71–0.98, *p*=0.032) No improvement of survival (43.9 m vs 44.8, HR: 1.01, 95% CI: 0.85–1.20, *p*=0.91)
[37]	CR M+/SRE	DEN 120 mg vs ZA 40 mg	↑ time to new SRE by 20.7 m with DEN vs 17.1 m with ZA (HR: 0.82, 95% CI 0.71–0·95; *p*=0.008)
[38]	CR M+/symptomatic skeletal events	DEN 120 mg vs ZA 4 mg	↓ risk for new SRE for DEN (HR, 0.78; 95% CI: 0.66–0.93; *p*=0.005) ↓ risk for any SRE for DEN (rate ratio: 0.78; 95% CI: 0.65–0.92; *p*=0.004)

## Abbreviations

BMD: bone mass density
FU: follow-up
NR: not reached
Doc: docetaxel
CS: castration-sensitive
ZA: zometa
HR: hazard ratio
CI: confidence interval
CPFS: clinical progression-free survival
QoL: quality of life
ADT: androgen-deprivation therapy
LN: lymph nodes
SOC: standard of care
SREs: skeletal-related events
CS: castration-resistant
OS: overall survival
PS: performance status
RTC: randomized control trials
DEN: denosumab
DT: doubling time.
